# Periodic Exposure of Keratinocytes to Cold Physical Plasma: An* In Vitro* Model for Redox-Related Diseases of the Skin

**DOI:** 10.1155/2016/9816072

**Published:** 2016-02-04

**Authors:** Anke Schmidt, Thomas von Woedtke, Sander Bekeschus

**Affiliations:** Leibniz-Institute for Plasma Science and Technology (INP Greifswald), ZIK plasmatis, Felix-Hausdorff-Strasse 2, 17489 Greifswald, Germany

## Abstract

Oxidative stress illustrates an imbalance between radical formation and removal. Frequent redox stress is critically involved in many human pathologies including cancer, psoriasis, and chronic wounds. However, reactive species pursue a dual role being involved in signaling on the one hand and oxidative damage on the other. Using a HaCaT keratinocyte cell culture model, we investigated redox regulation and inflammation to periodic, low-dose oxidative stress after two, six, eight, ten, and twelve weeks. Chronic redox stress was generated by recurrent incubation with cold physical plasma-treated cell culture medium. Using transcriptome microarray technology, we identified both acute ROS-stress responses as well as numerous adaptions after several weeks of redox challenge. We determined a differential expression (2-fold, FDR < 0.01, *p* < 0.05) of 260 genes that function in inflammation and redox homeostasis, such as cytokines (e.g., IL-6, IL-8, and IL-10), growth factors (e.g., CSF2, FGF, and IGF-2), and antioxidant enzymes (e.g., HMOX, NQO1, GPX, and PRDX). Apoptotic signaling was affected rather modestly, especially in p53 downstream targets (e.g., BCL2, BBC3, and GADD45). Strikingly, the cell-protective heat shock protein HSP27 was strongly upregulated (*p* < 0.001). These results suggested cellular adaptions to frequent redox stress and may help to better understand the inflammatory responses in redox-related diseases.

## 1. Introduction

The skin protects against environmental assaults and is crucial for maintaining the redox homeostasis [1]. The redox state is determined by the balance between pro- and antioxidative processes involving reactive oxygen and nitrogen species (ROS/RNS) [2]. Excessive presence of ROS/RNS is associated with a number of pathologies of the skin. This includes cutaneous malignancies [3], chronic wounds [4], and psoriasis [5]. There were an estimated 3.45 million new cases of cancer and 1.75 million deaths from cancer in Europe in 2012 [6]. The incidence of chronic wounds varies [7] but is expected to affect about 6.5 million patients in the United States [8]. The prevalence of psoriasis ranges from 0% to 8.5% according to age and geographic region [9]. Yet, it is important to recognize the dual role of reactive species, also in the human skin [10]. At low concentrations, they serve as signaling molecules [11] while at higher concentrations they become increasingly cytotoxic [12].

Generally, oxidants can be of exogenous or endogenous origin. The former derive from, for example, ozone, ionizing and nonionizing radiation, cigarette smoke, or invasion of pathogens and their associated infections [13]. The latter spring from oxygen-metabolizing enzymatic reactions (oxidases) and the leakage of superoxide from mitochondria [14]. Upon stimulation, the inducible nitric oxide (NO) synthase contributes to RNS production by generating large amounts of NO for defense or signaling purposes [15], also in keratinocytes [16]. Disturbance of the physiological balance between formation and removal of ROS/RNS (oxidative stress) disrupts the accurate interplay between the affiliated cells [17]. However, most toxic effects are counterbalanced by the complex and finely tuned antioxidative defense system [18].

We here attempted to investigate the redox response of HaCaT keratinocytes over three months. Frequent exposure to reactive species was generated using cold physical plasma. Cold plasma has been shown to have therapeutic benefit in diseases of the skin, such as chronic wounds and psoriasis [19–21]. The advantage of cold physical plasma over single oxidative agents is that it provides a more complex mixture of reactive molecules. Plasma was used to mimic the frequent redox stress apparent in redox-related diseases on the one hand and to investigate the effect of its long-term use on the other. We hypothesized that the cellular responses would substantially differ in cells exposed to rather acute (up to two weeks) or chronic (up to twelve weeks) oxidative challenge. Understanding these differences may help to better understand the skin tissue response in pathologies that involve chronic redox stress. Several studies have investigated acute oxidative stress conditions in keratinocyte skin cell culture models [22–24]. In contrast to primary skin tissue,* in vitro* cultures obviously allow the investigation of cells with the same genetic background over long investigation times and under highly controlled conditions.

Following repeated redox challenge, we used transcriptome microarray technology to study the molecular mechanisms and to identify markers of cellular redox modulation in keratinocytes. Periodic redox stress was applied over 3 months and coincided with an altered gene expression linked to cell metabolism, inflammation, and general stress response. Our model system was designed to emphasize mild redox conditions rather than applying cytotoxic dosages. We were able to show distinct transcript profiles of cytokines, keratins, and growth factors as well as an imperative involvement of junctional proteins in redox adaptions. Collectively, the presented system-wide modifications suggest that the simultaneous alteration of multiple pathways provides an important in-depth transcriptome overview linking permanent plasma-evoked oxidative stress effects with cell responses and adaption mechanisms, conceivably having implications in redox-based diseases of the skin.

## 2. Material and Methods

### 2.1. Cell Culture and Cold Physical Plasma

Three million epithelial keratinocyte HaCaT cells [25] were cultured in 75 cm^2^ dishes using RPMI1640 cell culture medium supplemented with 8% bovine calf serum (Sigma, USA), 0.1 mg/mL penicillin/streptomycin, and 2 mM L-glutamine (Lonza, Switzerland). Cold physical plasma-treated cell culture medium was generated by exposure of 5 mL of fully supplemented medium to an atmospheric pressure argon plasma jet (60 s;* kINPen 09*; neoplas tools, Germany) [26]. Three standard liters per minute of argon (99.99%; Air Liquide, France) were used as feed gas. The plasma was generated by applying a voltage of 2–6 kV_pp_ with a frequency of 1.0–1.1 MHz to the central electrode. Three million HaCaT keratinocytes were exposed to 15 mL of plasma-treated medium twice a week and passaged once a week (Figure 2(a)). After two, six, eight, ten, and twelve weeks, cells were either subjected to microscopic experiments or RNA or protein isolation.

### 2.2. Flow Cytometry

Keratinocytes were loaded with 5 *μ*M of CM-H_2_DCF-DA or mitotracker deep red (MTDR; both life technologies, USA) according to the manufacturer's instructions. After exposure to plasma-treated medium, cells were trypsinized and green fluorescence was quantified using a* Gallios* flow cytometer (Beckman-Coulter, USA). For assessment of apoptosis, keratinocytes were plasma-treated and incubated for 18 h at 37°C. Etoposide (50 *μ*M) was used as positive control (BioVision, USA). Subsequently, cells were loaded with active caspase 3 detection reagent (Enzo, USA) according to the vendor's protocol. Keratinocytes were then trypsinized and subjected to flow cytometric evaluation. Data analysis was conducted using* Kaluza* software (Beckman-Coulter).

### 2.3. Transcriptomic Analysis

RNA was purified according to the manufacturer's instructions (Bio&Sell, Germany) and RNA quality was maximal (scores of 10 out of 10; data not shown) as evaluated using low-volume gel electrophoresis (Bioanalyzer 2010; Agilent, USA). For cRNA generation, RNA of two independent experiments was pooled for each sample. cRNA was conjugated to Cy3, and transcriptional analysis was carried out using microarray-based exon analysis (Agilent) as described before [27]. Briefly, samples were hybridized onto SurePrint G3 custom GE 8 × 60 K chips (OakLabs, Germany) for 17 h at 65°C in a hybridization oven (Agilent). After washing, fluorescence intensities were recorded using a gene chip scanner (Agilent). Background-corrected signal intensities were determined and processed using feature extraction software (Agilent). Normalization, statistical tests, clustering, and filtering methods were conducted using gene expression analysis software (Partek, USA). Microarray data were deposited into gene expression omnibus database (GSE4876). Data were grouped according to their respective treatment regime (2, 6, 8, 10, and 12 weeks) and analyzed statistically using multiple testing corrections to identify differentially expressed genes (at least ±2-fold with *p* < 0.05). Also expression changes of less than ±2-fold are shown if target expression at one or more time points was greater than ±2-fold. Additionally, sets of coregulated genes were functionally clustered, and their biological relevance was analyzed using PANTHER software.

### 2.4. Protein Analysis

Instead of validating a large number of microarray results, we rather chose protein targets based on their significance within main cellular responses. These included oxidative defense (PRDX2 was among the 333 always differentially expressed genes (Figure 2(c)); HSP27 and its phosphorylated form were previously found to be central, studying the redox response in THP-1 monocytes [28]; NQO1 was also found to be redox-controlled using cold plasma [27]), cellular damage and apoptosis (histone H2AX and its phosphorylation are prominent markers of genomic DNA damage; Puma [29] and p21 [30] are involved in redox-related apoptosis induction), and cellular structure (keratin 1 showed the strongest regulation among all keratins (Table 2); *β*-actin served as housekeeping protein). For protein isolation, cells were lysed in RIPA buffer containing protease and phosphatase inhibitors (cOmplete Mini, phosSTOP; Roche, Germany) and 2 mM phenylmethanesulfonylfluoride (Roth, Germany). For western blotting, proteins of cell lysates were separated by sodium dodecyl sulfate polyacrylamide gel electrophoresis on precast 10% gels (AbCam, UK) and transferred to PVDF membranes (Roth). Immunoblots of whole protein extracts (20 *μ*g) were performed with antibodies against Puma and E-cadherin (all Cell Signaling, USA); occludin and ZO-1 (both life technologies, USA); and CTNNB1, *β*-actin, Hsp27, phospho-Hsp27, PRDX2, H2AX, and phospho-H2AX (all Santa Cruz, USA). Antibody binding was followed by three washing steps and incubation with horseradish peroxidase-coupled secondary antibodies before chemiluminescence (Light Polaris; Serva, Germany) detection (ImageQuant; GE Healthcare, USA). Band intensities were quantified using ImageQuantTL Software (GE Healthcare), normalized to total protein level, and expressed as fold change compared to the corresponding control.

### 2.5. Cell Migration and Immunofluorescence Microscopy

One million HaCaT keratinocytes were seeded onto 60 mm plastic culture dishes and incubated overnight to permit cell adhesion. Scratches were performed using a 10 *μ*L pipette tip. Gap closure was followed by time lapse microscopy (Zeiss, Germany) and three gap distances per samples were evaluated at different time points using* Axio Vision* software (Zeiss). For immunofluorescence, HaCaT keratinocytes were grown on glass coverslips for 24 h prior to fixation (4% paraformaldehyde; Sigma) and permeabilization (PBS with 0.1% Triton X-100; Sigma). Samples were subsequently incubated with primary antibodies (1 : 500; cell signaling technologies, USA) targeted at zona occludens protein 1 (ZO-1) overnight at 4°C. Cells were washed twice and incubated with appropriate Alexa Fluor 488-conjugated secondary antibodies (1 : 700; life technologies) for 1 h. Coverslips were washed and mounted onto glass microscope slides using mounting medium containing DAPI (VectaShield; Biozol, Germany) prior to analysis using an Axio Observer Z.1 (Zeiss).

## 3. Results

### 3.1. Cold Plasma Induced Redox Changes but Only Modestly Impacted Cell Viability

**Figure 1:**
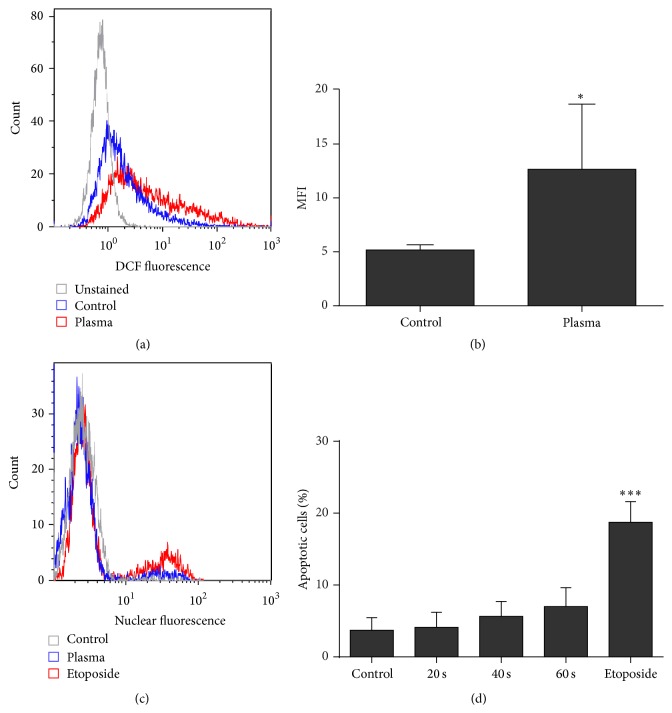
Exposure to plasma-treated medium evoked acute oxidative stress but was not cytotoxic. HaCaT keratinocytes were loaded with CM-H_2_DCF-DA and exposed to plasma-treated medium (60 s). Cells were subsequently trypsinized and fluorescence was measured using flow cytometry (a). Compared to control cells, intracellular mean fluorescence intensity (MFI) of DCF was significantly increased in treated keratinocytes (b). To assess viability, keratinocytes were exposed to plasma-treated medium and incubated for 18 h. Cells were stained for active caspase 3 and analyzed by flow cytometry (c). The number of apoptotic cells was significantly increased in etoposide control but not in cells exposed to plasma-treated medium (d). Data are presented as one representative (a, c) or mean + SD (b, d) of two independent experiments. Statistical comparison was done using* Student's t*-test (b) or one-way ANOVA with* Dunnett* corrections for multiple comparison to untreated control (d) (^*∗*^ *p* < 0.05, ^*∗∗∗*^ *p* < 0.001).

Plasma generates reactive species of various kinds and we found a fluorescence increase of intracellular DCF indicative of redox stress (Figures 1(a) and 1(b)). Then, we investigated cell viability after single application of plasma-treated medium. In contrast to positive control (etoposide), we identified an only subtle, nonsignificant increase in apoptotic cells receiving plasma-treated medium (Figures 1(c) and 1(d)). Thus, the plasma treatment time (60 s) was mediating a rather nontoxic oxidative challenge to keratinocyte cells.

### 3.2. HaCaT Keratinocyte Gene Expression Was Affected by Periodic Redox Challenge

**Figure 2:**
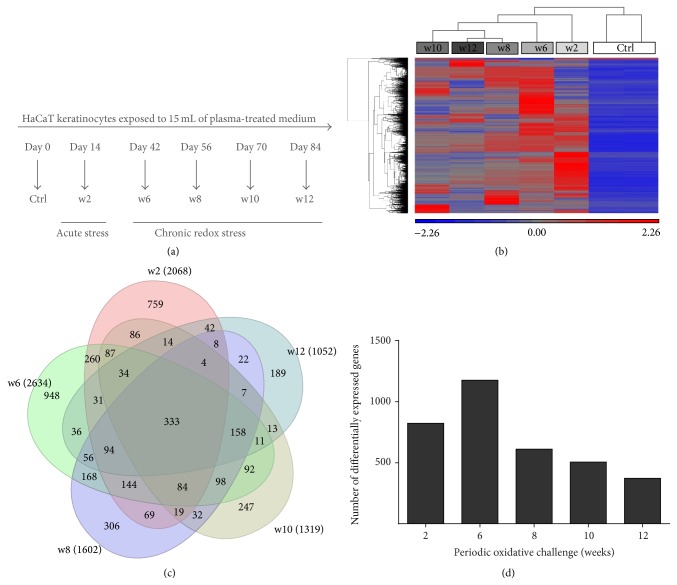
Repeated redox challenge modulated HaCaT keratinocyte gene expression profile. HaCaT keratinocytes were exposed to plasma-treated (60 s) medium twice per week, and gene expression was determined after acute (w2) and chronic (w6, w8, w10, and w12) exposure to redox stress (a). Hierarchical clustering of differentially expressed genes (upregulated: red; downregulated: blue) after several weeks of plasma-mediated redox challenge (w2 to w12) is shown (b). The Venn diagram visualizes the overlap of the differentially expressed genes (number in parentheses) between different groups (in color) compared to untreated HaCaT keratinocytes (c). Numbers of differentially expressed genes with unambiguous identification are given for each treatment regimen and according to PANTHER classification (d).

**Figure 3:**
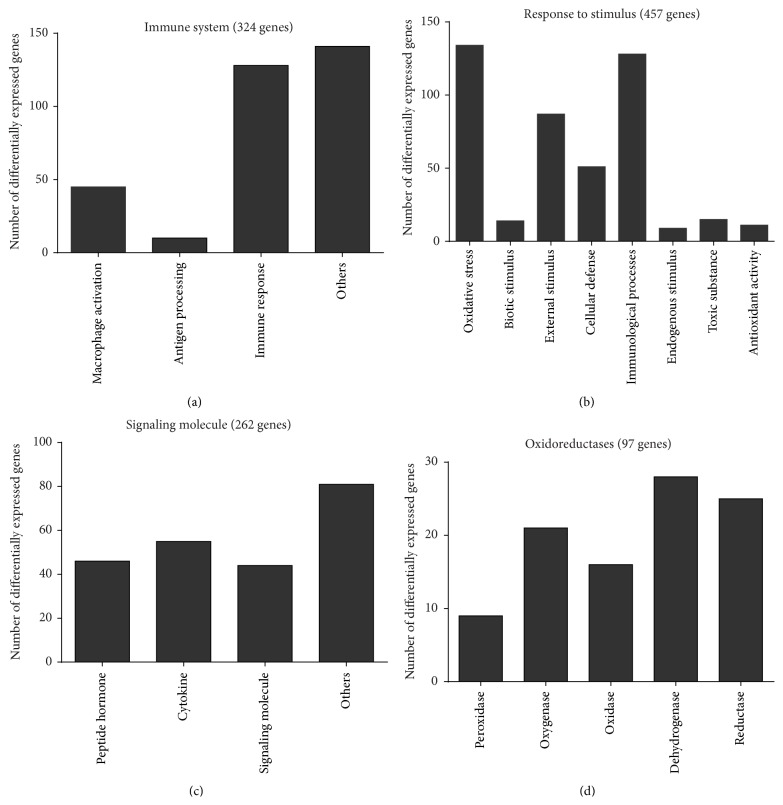
Gene ontology (GO) classification of the identified genes differentially expressed. PANTHER classification system was used to analyze the gene lists for each experimental group (w2–w12 versus untreated control) to list the categories with the “biological process” functions domain of GO (*p* < 0.05). Differentially expressed genes in the categories “immune system” (a) and “response to stimulus” (b) are shown. Top hits of the protein classes associated with the repeated redox challenge include signaling molecules (c) and oxidoreductases (d). The total number of modulated genes is given in parentheses.

Next, we repeatedly exposed HaCaT keratinocytes to cold physical plasma-treated (60 s) cell culture medium over three months to assess its impact on global gene expression (Figure 2(a)). Hierarchical clustering illustrated the statistically relevant changes in gene expression between oxidatively challenged compared to untreated cells (Figure 2(b)). We detected a total of approximately 3,000 genes with at least ≥2-fold increase or decrease of expression. Venn diagrams were constructed to identify the number of exclusively up- or downregulated genes corresponding to the different sampling time points. Overlapping differences shared among more than one sample group comparison are represented in the areas of intersection between two or more circles. The Venn diagram showed the overlapping genes among the 2,068, 2634, 1602, 1319, and 1052 transcripts differentially expressed between the w2 and w12 groups (Figure 2(c)). The 333 transcripts in the center of the Venn diagram represent genes that are differently expressed among all treatment groups (in contrast to control). A total of 123 genes with ID classification (from these 333 transcripts) were identified using PANTHER software (Supplemental Table 1A) (see Supplementary Material available online at http://dx.doi.org/10.1155/2016/9816072) and were further subdivided into several functional and protein classes (Supplemental Table 1B). In total, 411, 1079, 709, 453, and 321 transcripts were upregulated, and 496, 1241, 850, 527, and 391 transcripts were downregulated in our different groups from w2 to w12 and in comparison to untreated keratinocytes, respectively. In comparison to w8–12, the number of differentially expressed genes was higher at the relatively earlier time points (w2 and w6) (Figure 2(d)). The differentially expressed genes with a known function were assigned to gene ontology (GO) and protein classes (Figure 3). Significant GO terms (*p* < 0.05; PANTHER database) were “immune system” (324 genes) and “response to stimulus” (457 genes) (Figures 3(a) and 3(b)). Within the latter, a large number of genes were involved in “stress responses” (134 genes) and “immunological processes” (128 genes). Further, there were a number of genes related to signaling (Figure 3(c)), such as protein kinases, phosphatases, growth factors, cytokines, and chemokines. We also identified a large set of genes belonging to oxidoreductases (Figure 3(d)). Taken together, the transcriptomic investigation revealed a number of modulations following cyclic oxidative stress.

### 3.3. The Antioxidative Defense System Was Activated by Frequent Redox Stress

**Figure 4:**
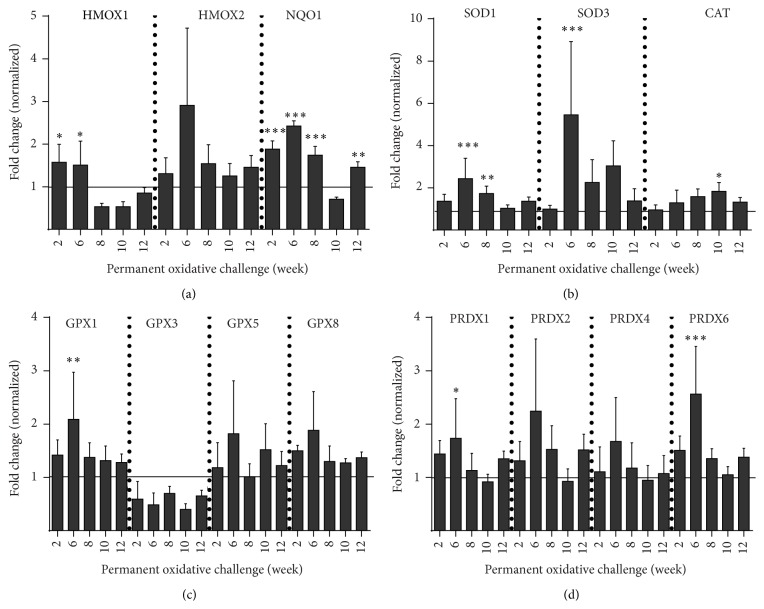
Transcriptional response of the antioxidant defense system to acute and chronic redox challenge. Transcription of several antioxidative enzymes in HaCaT keratinocytes was measured using microarray technology. HMOX2 and NQO1 were mainly upregulated over three months in contrast to a transient upregulation of HMOX1 during acute stress phases followed by downregulation after chronic stress exposure (a). SOD1, SOD3, and catalase were mainly upregulated (b). GPX1, GPX5, and GPX8 mRNA copy numbers were enhanced whereas those of GPX3 were found to be reduced (c). Expression of several peroxiredoxins was increased after periodic oxidative stress (d). Data are presented as mean + SD of two analyses. Statistical analysis was done using one-way ANOVA with* Dunnett* corrections for multiple comparisons to untreated, normalized control (^*∗*^ *p* < 0.05, ^*∗∗*^ *p* < 0.01, ^*∗∗∗*^ *p* < 0.001).

We extended our transcriptomic view to enzymes involved in resolving redox stress (Figure 4). Stress signaling on downstream factors, such as heme oxygenases (HMOX) and NADPH quinone oxidoreductase 1 (NQO1), was at least in part significantly increased (Figure 4(a)). Initial (week 2) superoxide dismutases 1 and 3 (SOD1/3) expression was unaffected whereas their expression reached a maximum at week 6 of periodic oxidative challenge (Figure 4(b)). Expression of catalase was moderately but significantly increased after ten weeks of frequent plasma exposure. Glutathione peroxidases (GPX1, GPX5, and GPX8) showed an overall modest upregulation while GPX3 was found to be constantly downregulated (Figure 4(c)). Peroxiredoxin (PRDX) 1, 2, 4, and 6 expressions were rather enhanced, especially after week 6. A significant increase of expression of members of the antioxidative defense system was most prominent after 6 weeks of frequent exposure to plasma-treated medium (Figure 4(d)), indicating strong adaption responses of keratinocytes.

### 3.4. Apoptosis Pathways

**Figure 5:**
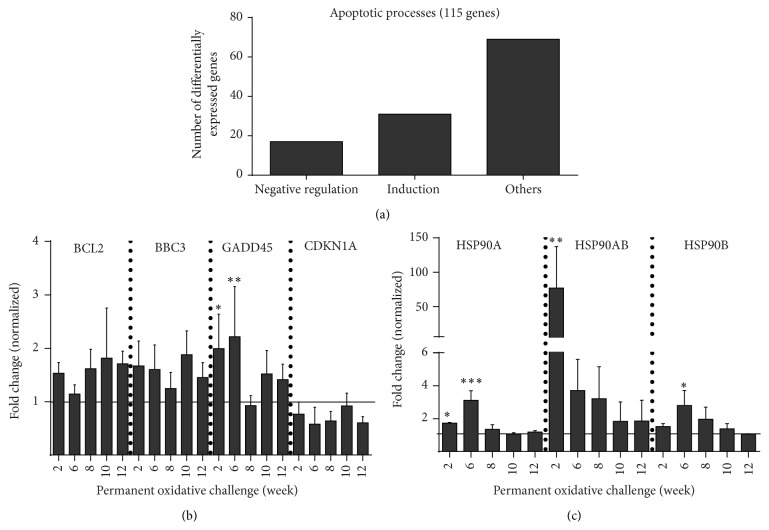
Apoptosis-related gene expression in response to periodic redox challenge. One hundred and fifteen apoptosis-related genes were associated with frequent redox challenges whereas 17 and 31 genes belonged to negative or positive regulation apoptosis, respectively (a). Downstream targets of p53 signaling pathway included BCL2 and BBC3 which represent the proapoptotic p53 pathway and GADD45 that is involved in DNA repair. All of them were mainly upregulated. CDKN1A plays a role in cell cycle control and senescence and was slightly downregulated (b). Expression of stress-related heat shock proteins was partially enhanced as well (c). Data are presented as mean + SD of two analyses (b, c). Statistical analysis was done using one-way ANOVA with* Dunnett* corrections for multiple comparisons to untreated, normalized control (^*∗*^ *p* < 0.05, ^*∗∗*^ *p* < 0.01, ^*∗∗∗*^ *p* < 0.001).

**Figure 6:**
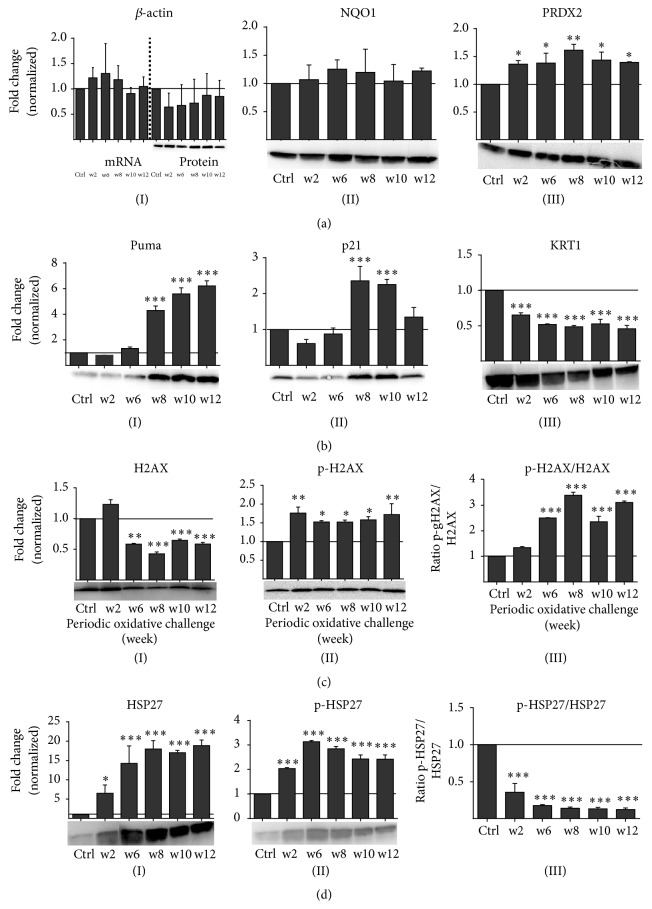
Western blot analysis showed a differential expression of several redox-related proteins. Western blot analysis confirmed the RNA expression profiles of *β*-actin ((a)(I), no change) and of the antioxidative enzymes NQO1 ((a)(II)) and PRDX2 ((a)(III)). Puma and p21 ((b)(I), (b)(II)) were upregulated after chronic but not acute oxidative challenge whereas keratin 1 was downregulated ((b)(III)). The total amount of H2AX protein was twofold downregulated after periodic oxidative challenge in contrast to acute stress ((c)(I)) while its phosphorylated form *γ*-H2AX was slightly upregulated in response to oxidative stress ((c)(II)). The ratio of p-H2AX to the total protein amount of H2AX was significantly enhanced after chronic oxidative challenge ((c)(III)). The total amount ((d)(I)) and the phosphorylated form ((d)(II)) of the stress-related protein HSP27 was significantly enhanced after repeated plasma treatment. The ratio of p-HSP27 to total protein amount of HSP27 was significantly decreased ((d)(III)). Representative western blot images are shown. Data in diagrams are presented as mean + SD of two independent experiments. Statistical analysis was performed using one-way ANOVA with* Dunnett* corrections for multiple testing to untreated, normalized control (^*∗*^ *p* < 0.05, ^*∗∗*^ *p* < 0.01, ^*∗∗∗*^ *p* < 0.001).

Only a small number of genes involved in apoptotic signaling were found in our transcriptomic studies with 31 genes being positive and 17 genes being negative regulators of apoptosis (Figure 5(a)). BCL2 and BBC3 (both p53-upregulated inducers of apoptosis) transcript levels were only nonsignificantly increased throughout the three months of treatments. Similarly, downregulation of the cell cycle gate-keeper CDKN1A (p21) was steady but modest (Figure 5(b)) while upregulation of the DNA repair enzyme GADD45 was significant at w2 and w6. Heat shock proteins protect cells from excessive protein stress and subsequently from apoptosis, and we found several candidates (HSP90A, HSP90AB, and HSP90B) to be significantly upregulated at w2 and/or w6 (Figure 5(c)). In contrast, actin mRNA and protein expression remained relatively unchanged (data not shown). Therefore, *β*-actin was used as loading control in western blot analysis (Figure 6(a)(I)). Correlating to transcriptomics, western blot analysis of the antioxidative enzymes NQO1 and PRDX2 confirmed a slight or significant upregulation, respectively (Figures 6(a)(II) and 6(a)(III)). Contrasting data on mRNA expression, a strong upregulation was seen for Puma (BBC3) and p21 (CDKN1A). Their protein level remained significantly elevated at weeks 8 to 10 of cyclic oxidative challenges with p21 but not Puma decreasing after that (Figures 6(b)(I) and 6(b)(II)). For H2AX, we found a twofold repression of its total protein (Figure 6(c)(I)) amount whereas its phosphorylated form (*γ*-H2AX) and the ratio between phosphorylated und total protein were increased (Figures 6(c)(II) and 6(c)(III)), indicating cellular perception of redox stress. Moreover, we detected a significant increase of the cytoprotective HSP27 total protein as well as its phosphorylated form throughout this study from w2 to w12 (Figures 6(d)(I) and 6(d)(II)). The p-HSP27/HSP27 ratios negatively correlated to number of passages periodically receiving oxidative challenge (Figure 6(d)(III)).

### 3.5. Gene Expression of Structural Proteins Was Strongly Affected with Frequent Redox Stress

Structural proteins serve as important barriers and mediate cell-cell contact. We were able to identify a differential expression of occludin (OCLN), several claudins (CLDN 1–4, 7, 8, 12, 17–19, 22, and 23), and zonula occludens (ZO) tight junction proteins (ZO-1, ZO-2, and ZO-3) (Table 1). Interestingly, ZO-1 protein was only upregulated after initial redox challenges up to 6 weeks. CDHs are linked to actin via *β*-catenin (CTNNB; cadherin-associated protein), and mRNA expression of the latter was also enhanced following repeated exposure to redox stress. We also discovered transcriptional changes in a striking number of keratins (KRT) and their associated proteins (KRTAP) as well as matrix-metalloproteinases (MMPs) and other structural proteins (Table 2). A significant decline was seen with KRT1, KRT4, KRT13, and KRT77 while others were strongly upregulated (e.g., KRT35, KRT38, KRT72, KRT82, and KRT83) after redox stress. KRT1 is a major epithelial keratin and western blotting confirmed its significantly lower expression after periodic oxidative challenge (Figure 6(b)(III)). The MMPs investigated seemed to be always upregulated in oxidative stress conditions. While acute stress only modulated few targets investigated (20/66 in w2), chronic stress affected a larger number of transcripts (47/66 in w6) with declining numbers after that (32/66 in w8; 31/66 in w10; 21/66 in w12).

### 3.6. Redox Stress Reduced HaCaT Keratinocyte Motility and Induced Morphological Changes

**Figure 7:**
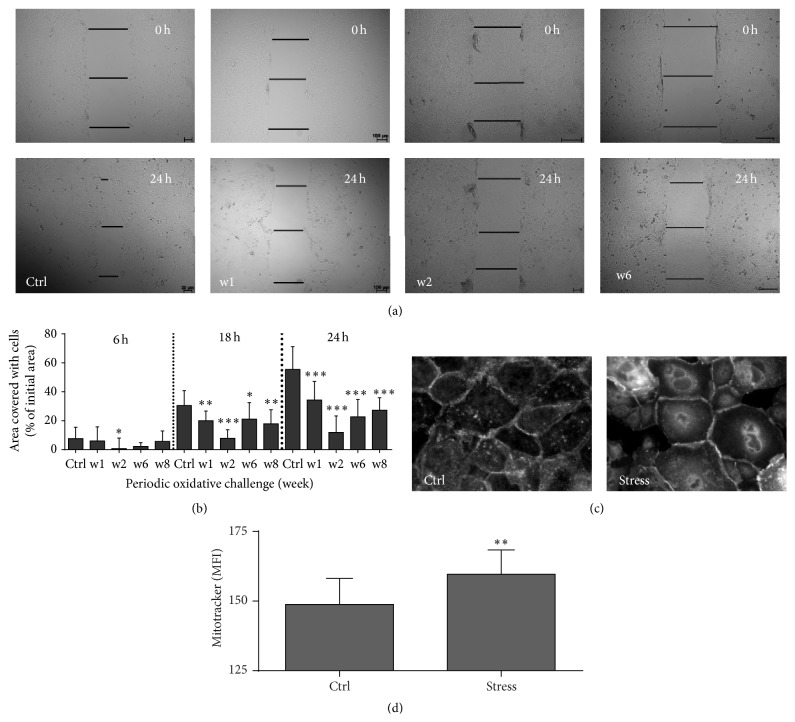
Periodic oxidative redox challenges decelerated keratinocytes motility and migration. Scratch assays were performed to monitor artificial wound healing over 24 h. Representative images are shown for controls and repeatedly (1–8 weeks) plasma-treated cells (a). There was a significant decrease in cell motility following plasma induced redox stress (b). Compared to control cells (ctrl) plasma-treated cells (w2) displayed a notable enlargement in size as seen after staining cell margins for ZO-1 (c). Mitotracker red fluorescence (MFI), which is a marker for total mitochondrial content, increased after chronic stress and was measured using flow cytometry (d). Data are presented as mean + SD of two to four experiments and statistical comparison was done using ANOVA with* Dunnett* correction for multiple comparison to untreated, normalized control (b), or* Student's t*-test (d) (^*∗*^ *p* < 0.05, ^*∗∗*^ *p* < 0.01, ^*∗∗∗*^ *p* < 0.001).

Scratch assay was performed to determine keratinocyte motility using time lapse video microscopy (Figure 7(a)). Repeated plasma treatment significantly repressed keratinocyte motility following oxidative stress (Figure 7(b)). Interestingly, repression peaked at w2 but was partially reversed after further redox challenges (w6 and w8). Visually, periodic exposure to oxidative stress apparently enlarged cell size as shown using immunofluorescence staining with ZO-1 (Figure 7(c)). Cell size enhancement may be linked to a higher number of mitochondria. We therefore assessed the total mitochondrial content. Mitotracker fluorescence was measured in periodically plasma-treated cells and compared with untreated keratinocytes. Using flow cytometry, a moderate but significant increase in mitochondrial content in stressed cells supported the notion of enlarged cell bodies after oxidative stress challenge (Figure 7(d)).

### 3.7. Plasma-Treated Medium Elicited a Distinct Secretory and Inflammatory Profile

Secretory products, such as cytokines, growth factors, and other inflammatory mediators, serve to mediate autocrine and paracrine communication between cells. An overview of the altered cytokine expression is given (Table 3). Cold plasma induced not only proinflammatory (e.g., IL-4, IL-10, and TGF*β*) but also anti-inflammatory (e.g., IL-6, IL-8, and TNF*α*) cytokines. Moreover and in response to plasma-mediated oxidative stress, HaCaT keratinocytes contained significantly increased levels of several growth factor transcripts (e.g., CSF2, GAS1, FGF6, and IGFs).

## 4. Discussion

We investigated the HaCaT keratinocyte global transcriptomic profile over three months to identify genes reflecting adaptions to periodic oxidative stress as seen in redox-related diseases of the skin. The frequent redox challenge was applied using cold physical plasma-treated medium as in previous proteomics studies [31]. Its use is advantageous over addition of single oxidants as it provides a complex mixture of reactive components that either originate directly from the plasma gas phase or are formed during secondary reactions with ambient air or water molecules [32]. This includes the deposition of biologically active reactive molecules, such as superoxide, hydroxyl radicals, nitrite, nitrate, peroxynitrite, and hydrogen peroxide [33–35]. Accordingly, and in line with previous results using other cell types [36–38], plasma-treated medium led to intracellular oxidation (Figure 1). It is important to consider the dual role exhibited by ROS and RNS [39]. While cytotoxic effects occur at high doses [40], low-dose redox stimulation is imperative in cell physiology [41]. Activation of kinases and inhibition of phosphatases are critically regulated by reactive species [42]. Reactive species also play a crucial role in wound healing [43]. In particular hydrogen peroxide is an important second messenger for HaCaT keratinocyte cell growth [44]. Cellular RNS are mainly produced secondary to signaling events initiated upon ROS [45]. We here present a group of interesting targets in response to repetitive redox stress.

Insufficient removal of reactive intermediates leads to intracellular accumulation of ROS and results in oxidative stress [46–48]. This may result in induction of apoptosis and p53 activation through phosphorylation of MAP kinases and activation of Bcl-2 family proteins [49–51]. Within the transcriptome, our continuous plasma treatment targeted apoptotic processes in HaCaT cells only to a minor extent. By contrast, Puma and p21 protein were upregulated from w6 to w12, indicating a differential apoptotic programming with long-term redox challenges [52, 53]. GADD45 modifies DNA-accessibility if damage is anticipated and its upregulation was only transient (w2 to w6) suggesting that adaptive mechanisms take place with frequent redox challenges. The ROS/RNS-associated, antiapoptotic heat shock proteins are mediators between p53 and mitogen-activated protein (MAP) kinase signaling [54]. The cell stress chaperone HSP90, which is essential for protein folding and transports within the cell [55], and both total HSP27 protein and its phosphorylated form, which function in signal transduction and inhibition of apoptosis [56], were significantly upregulated during frequent oxidative challenge. Both molecules are promising clinical targets to reduce oxidative stress [57, 58]. H2AX facilitates recruitment of DNA repair proteins [59] and becomes phosphorylated (*γ*-H2AX) as a downstream effect of the apoptosis cascade and in response to DNA replication stress in a ATR-dependent manner [60]. The increased presence of *γ*-H2AX suggests that genomic stress was perceived throughout the 3 months of treatment. We could not find a significant modulation of ATR expression (data not shown) which corroborates the results of a previous study investigating the transcriptome in cold plasma-treated corneas [61].

Oxidative or nitrosative stress is usually counteracted by enzymatic scavengers while silencing of antioxidative defense enzymes enhances cellular damage and cancer formation [62]. We found an upregulation of several peroxiredoxins and glutathione peroxidases except for GPX3 (Figure 4). GPX1, GPX5, and GPX8 prevent peroxide-induced oxidative damage, lipid peroxidation, and protein degradation [63–65]. They were upregulated rather at the beginning of chronic oxidative challenge (w6) suggesting an adaption of keratinocytes within the antioxidative defense system during the later course of culture. The upregulation of PRDX2, another important antioxidative enzyme, has also been found in psoriasis [66]. The downmodulation of GPX3, the only extracellular member of the glutathione peroxide family [67], may have been compensated by the concomitant upregulation of the other antioxidative enzymes. The overall upregulation of the antioxidative system corroborates our recent results involving a redox-based activation of the Nrf2 pathway controlling HMOX1 expression [27]. Our results also correlate well with previous studies that identified a subtle decrease of HMOX1 but not HMOX2 [68] following redox stress.

Keratinocytes appear to play an important role in differentiation, migration, and reepithelialization in the final stage of wound closure [69]. For acute redox stress, plasma-treated medium notably repressed cell migration which is in agreement with our previous results using a carcinoma cell line [70]. Interestingly, cell migration activity partly recovered afterwards, pointing to an adaption of keratinocytes to periodic redox challenges. Proliferation is tightly linked to cell-cell contacts and adhesion molecules, such as tight junctions [71–73]. Downregulation of junctional proteins (e.g., occludins and claudins) is often associated with impairment of barrier function and disease [74–76]. Several of such transcripts were modulated by repetitive redox challenges, possibly indicating a reorganization of the junctional network in response to ROS as also suggested by others [77–79]. Keratins also contribute to the barrier properties [80] and are associated with regulatory functions [81], forming a signaling network with kinases [82]. A large number of keratin transcripts were differentially expressed following repeated exposure to oxidative mediators, possibly being responsible for the observed differences in cellular migration. Moreover, they may be linked to the observed increase in cell size. This corroborates our previous findings in plasma-treated THP-1 cells [83] and links to ROS-mediated increase in keratinocyte cell size and differentiation [84]. In support of this notion, LCE1A, which is expressed in late stages of keratinocyte maturation [85], was significantly upregulated. Filaggrin (FLG), another important component of protective skin layers of the epidermis was downregulated. A loss of FLG function is associated with several skin diseases such as* ichthyosis vulgaris* [86] and atopic dermatitis [87]. Both LCE1A and FLG belong to the cornified envelope which was recently shown to be highly involved in redox regulation via ROS quenching [88].

We identified a number of secretory factor transcripts being regulated via frequent redox stress. The extracellular matrix is important for keratinocyte migration in wound healing but also prone to MMP digestion [89]. As such, MMPs participate in physiological (e.g., angiogenesis and wounds healing) and pathological (e.g., cancer and nonhealing wounds) processes [90–92]. MMP2 (collagenase IV) and MMP16 (activates MMP2) expression was significantly upregulated at all time points, suggesting a detrimental role in wound healing processes [93]. Moreover, we found a differential regulation of transcripts associated with inflammatory processes in the skin (e.g., S100A) [94], which are associated with an altered cellular phenotype, and enhanced expression of inflammatory mediators, for example, cytokines [95]. Such immune mediators are often dysregulated in chronic wounds [96]. IL-19 transcript numbers were increased in response to periodic redox stress and were previously reported to upregulate the expression of proinflammatory IL-6 and TNF*α* which together with IL-1 and IL-8 were also found to be upregulated in our study [97]. These mediators were shown to be elevated in nonhealing wounds [98–101]. Also highly proinflammatory neutrophils are chemotactic for CSF2 [102], and in line with previous studies [103] CSF2 was found to be constantly elevated in response to redox stress. However, periodic redox stress also increased the transcription of anti-inflammatory mediators. IL-37 is a fundamental inhibitor of innate immunity [104] and keratinocyte-derived IL-4 and IL-10 mediate immune suppression [105]. TGF*β* has been used in clinical trials combating deficient wound healing [106] and was partially increased following plasma as well. Frequent redox challenges thus generated not only a pro- but also anti-inflammatory cytokine signature and thus may have a complex impact on the quality of inflammation in redox-related diseases.

We can only speculate why there was no consistent up- and downregulation patterns over time in the targets investigated in this study. First, gene expression may be heterogeneous within cultured cells. Frequent cold plasma treatment may have promoted growth of cells that are better equipped against oxidative stress, consequently overgrowing the cells that are not. For example, single cold plasma treatment of keratinocytes led to G2/M-phase cell cycle arrest but this was dependent on the total treatment time and was not present in all cells [107]. Alternatively, all keratinocyte cells may have adapted to redox stress over time, significantly altering their basal gene expression profile.

## 5. Conclusion

Using cold physical plasma-derived reactive species, our keratinocyte-based* in vitro* model aimed to mimic the characteristics of chronic oxidative stress in redox-related disorders of the skin to pinpoint biomarkers for molecular therapies. Redox processes are important decision makers in skin disease and tumorigenesis and here affected cell-cell communication, cellular proliferation, and inflammatory processes. The identification of genes and proteins whose expression is altered following permanent oxidative challenge is an important step to better understand redox regulations in clinical settings.

## Supplementary Material

Supplementary Table: Differentially expressed genes present in all groups investigated (A)and their classification into protein functions and classes (B).

## Figures and Tables

**Table 1:** Expression (fold change compared to untreated control) of tight and adherent junction proteins with at least one significant modulation throughout the time course of plasma treatment. Actin expression remained relatively unchanged.

Gene name	Gene ID	w2	w6	w8	w10	w12
Occludin	OCLN	1.32	−1.15	**−2.11**	**−2.02**	−1.86

Claudin 1	CLDN1	−1.11	**−2.32**	−1.38	−1.01	1.04
Claudin 2	CLDN2	**2.17**	**2.11**	1.40	1.48	−1.26
Claudin 3	CLDN3	1.97	1.68	**−4.12**	**−2.41**	−1.27
Claudin 4	CLDN4	1.66	**3.19**	−1.02	1.00	1.08
Claudin 6	CLDN6	1.59	**2.52**	−1.16	1.37	**−1.99**
Claudin 7	CLDN7	1.07	−1.38	**−2.03**	−1.56	−1.08
Claudin 8	CLDN8	1.05	**−10.46**	−1.86	−1.31	1.06
Claudin 12	CLDN12	1.42	**2.21**	1.67	1.78	1.28
Claudin 17	CLDN17	1.86	−1.70	−1.93	**−2.66**	**−2.61**
Claudin 18	CLDN18	**4.86**	−1.11	−1.27	−1.74	−1.81
Claudin 19	CLDN19	1.86	**−2.46**	**−2.41**	−1.04	1.38
Claudin 22	CLDN22	**17.47**	**2.93**	−1.66	1.26	−1.52
Claudin 23	CLDN23	−1.24	−1.68	**−3.49**	**−2.51**	−1.69

Zonula occludens protein 1	TJP1 (ZO-1)	1.68	**3.06**	1.14	−1.09	1.10
Zonula occludens protein 2	TJP2 (ZO-2)	−1.05	**−2.27**	**−2.26**	−1.53	−1.07
Zonula occludens protein 3	TJP3 (ZO-3)	−1.62	**−2.60**	−1.45	−1.08	−1.39

Cadherin 2	CDH2	**4.78**	**46.49**	**11.74**	1.56	6.26
Cadherin 3	CDH3	1.37	**2.10**	1.70	1.26	1.25
Cadherin 4	CDH4	1.66	**3.19**	−1.02	1.00	1.08
Cadherin 5	CDH5	1.36	**2.06**	−1.06	1.78	1.15
Cadherin 7	CDH7	1.85	1.97	1.32	**2.29**	**2.27**
Cadherin 9	CDH9	**2.53**	**−3.54**	**−3.35**	**−2.47**	**−2.51**
Cadherin 10	CDH10	**2.14**	1.32	1.31	1.63	1.20
Cadherin 13	CDH13	−1.19	**2.95**	**3.40**	**3.34**	−1.09
Cadherin 16	CDH16	**3.55**	−1.47	−1.42	−1.82	1.11
Cadherin 18	CDH18	**2.04**	−1.14	**2.48**	1.18	−1.02
Cadherin 19	CDH19	**2.32**	−1.35	−1.34	−1.35	−1.31
Cadherin 20	CDH20	**5.80**	1.76	**2.35**	1.67	−1.14
Cadherin 23	CDH23	**3.07**	1.02	**2.48**	1.47	1.90
Cadherin 24	CDH24	1.53	**2.83**	1.61	1.25	1.06

*α*-actin 2	ACTA2	1.10	−1.21	**2.70**	1.41	1.06
*β*-actin	ACTB	1.34	1.35	1.23	1.04	1.02
*β*-catenin 1	CTNNB1	1.62	**2.05**	1.27	1.03	1.21

**Table 2:** Expression (fold change compared to untreated control) of matrix-metalloproteinases and fibrous structural proteins (keratins) with at least one significant modulation throughout the time course of plasma treatment.

Gene name	Gene ID	w2	w6	w8	w10	w12
Matrilysin	MMP7	**53.36**	**3.49**	**3.41**	**2.65**	**2.34**
Matrix metallopeptidase 27	MMP27	**3.00**	**4.16**	**3.81**	**2.74**	**3.16**
Stromelysin 2	MMP10	**2.08**	**124.31**	**2.81**	**2.10**	**2.27**

Keratin I cytoskeletal 13	KRT13	−1.22	**−5.77**	−1.46	−1.35	−1.03
Keratin I cuticular Ha5	KRT35	1.43	**12.00**	1.71	1.61	1.20
Keratin I cuticular Ha8	KRT38	**12.15**	**3.24**	**11.82**	**2.67**	1.25
Keratin II cytoskeletal 1	KRT1	−1.21	**−87.56**	**−2.72**	−1.57	1.13
Keratin II cytoskeletal 1b	KRT77	−1.78	**−41.78**	**−4.12**	**−2.51**	−1.65
Keratin II cytoskeletal 4	KRT4	1.20	**−3.27**	−1.20	−1.15	−1.03
Keratin II cytoskeletal 8	KRT8	1.46	**3.42**	1.68	1.26	1.28
Keratin II cytoskeletal 72	KRT72	**5.70**	**3.19**	**3.04**	**2.23**	**3.78**
Keratin II cytoskeletal 79	KRT79	−1.08	−1.87	−1.63	**6.24**	−1.56
Keratin II cuticular Hb2	KRT82	1.35	**2.86**	**2.74**	1.94	**3.03**
Keratin II cuticular Hb3	KRT83	1.79	**2.77**	1.18	−1.24	1.28
Keratin-associated 3-3	KRTAP3-3	**5.60**	1.42	**2.04**	1.48	−1.01
Keratin-associated 4-1	KRTAP4-1	−1.18	−1.12	**5.29**	−1.11	1.21
Keratin-associated 4-2	KRTAP4-2	−1.10	**−2.06**	1.76	**11.54**	**−2.28**
Keratin-associated 10-7	KRTAP10-7	−1.76	**3.76**	**2.81**	**3.08**	**3.84**
Keratin-associated 10-10	KRTAP10-10	−1.11	**−2.03**	**3.13**	−1.62	−1.13
Keratin-associated 10-11	KRTAP10-11	**5.66**	**3.17**	−1.40	−1.42	1.72
Keratin-associated 10-12	KRTAP10-12	−1.27	**8.01**	**4.78**	**2.68**	1.25
Keratin-associated 13-3	KRTAP13-3	1.19	**35.43**	−1.23	−1.26	1.54
Keratin-associated 16-1	KRTAP16-1	−1.22	−1.30	−1.20	**25.27**	1.49
Keratin-associated 22-2	KRTAP22-2	−1.29	**5.20**	−1.54	−1.54	1.89

Filaggrin	FLG	**−14.0**	1.05	−1.23	**−3.29**	−1.41
Laminin subunit alpha-1	LAMA1	**2.05**	**2.09**	1.79	1.31	1.19
Late CE protein 1A	LCE1A	1.71	**7.89**	**3.03**	**2.05**	1.65
Loricrin	LOR	−1.68	**−6.88**	**−2.35**	**−3.90**	−1.18
Protein S100-A4	S100A4	−1.44	**−4.27**	**−2.75**	−1.14	−1.17
Protein S100-A6	S100A6	−1.06	**−3.53**	−1.65	−1.53	−1.25
Protein S100-A7	S100A7	1.23	**−4.63**	**−4.54**	1.28	1.51
Protein S100-A8	S100A8	1.02	**−8.31**	**−3.31**	1.21	1.17
Protein S100-12	S100A12	−1.39	**−4.62**	**−2.42**	1.28	1.28

**Table 3:** Expression (fold change compared to untreated control) of cytokines and growth factors with at least one significant modulation throughout the time course of plasma treatment.

Gene name	Gene ID	w2	w6	w8	w10	w12
Interleukin-1*α*	IL1A	**11.85**	**17.09**	**4.01**	1.36	**3.18**
Interleukin-1*β*	IL1B	**4.30**	**5.65**	**2.30**	−1.01	**2.14**
Interleukin-4	IL4	**5.72**	−1.34	1.05	−1.33	−1.77
Interleukin-5	IL5	**5.51**	−1.06	**−2.05**	**−2.34**	−1.92
Interleukin-6	IL6	**3.38**	**3.95**	1.28	1.82	**3.48**
Interleukin-8	IL8	**2.25**	**2.83**	1.28	−1.84	−1.25
Interleukin-9	IL-9	−1.39	−1.12	**2.85**	−1.06	−1.26
Interleukin-10	IL10	**3.81**	1.68	**2.08**	1.36	1.15
Interleukin-11	IL-11	1.02	**−2.34**	−1.50	−1.39	−1.80
Pro-interleukin-16	IL16	**3.02**	−1.45	−1.42	1.98	1.79
Interleukin-17A	IL-17A	1.74	**2.74**	1.35	**2.56**	1.39
Interleukin-17B	IL17B	**13.90**	**3.20**	**2.94**	**2.07**	**7.54**
Interleukin-17D	IL17D	**2.91**	**11.47**	**3.16**	−1.41	1.55
Interleukin-18BP	IL-18BP	**−2.24**	1.12	1.41	1.46	−1.74
Interleukin-17F	IL17F	−1.46	**11.22**	−1.55	−1.09	−1.13
Interleukin-19	IL19	**2.09**	**2.51**	1.03	**2.05**	−1.32
Interleukin-24	IL24	1.16	**3.53**	**3.13**	**2.21**	**2.27**
Interleukin-31	IL31	1.85	**16.15**	**13.18**	**13.64**	1.49
Interleukin-32	IL32	**2.25**	1.82	−1.07	1.59	1.29
Interleukin-33	IL33	**2.87**	**2.23**	1.19	1.30	−1.70
Interleukin-34	IL34	**2.01**	**2.46**	−1.07	**2.39**	−1.20
Interleukin-36*α*	IL36A	**4.38**	1.70	−1.23	1.13	−1.01
Interleukin-36*β*	IL36B	**6.59**	**2.31**	**2.33**	1.25	**9.74**
Interleukin-37	IL37	1.17	**3.11**	1.36	−1.06	1.13

Colony stimulating factor 2	CSF2	1.69	**6.91**	**2.54**	1.71	1.67
Fibroblast growth factor 2	FGF2	**−3.02**	**2.57**	−1.24	1.80	1.36
Fibroblast growth factor 5	FGF5	**8.95**	−1.22	−1.16	1.03	**9.55**
Fibroblast growth factor 6	FGF6	1.58	1.20	**7.19**	1.45	**3.01**
Fibroblast growth factor 9	FGF9	−1.34	**3.59**	−1.51	1.77	**2.68**
Fibroblast growth factor 14	FGF14	1.13	−1.08	1.08	−1.06	**3.10**
Fibroblast growth factor 18	FGF18	−1.24	**13.76**	1.68	−1.09	−1.86
Fibroblast growth factor 21	FGF21	1.74	−1.40	−1.30	**6.29**	1.47
Fibroblast growth factor 22	FGF22	**3.02**	**3.03**	−1.33	−1.37	1.47
Growth arrest-specific protein 1	GAS1	**3.56**	**2.33**	1.97	1.86	**2.36**
Growth arrest-specific protein 2	GAS2	−1.22	−1.14	−1.03	**8.87**	1.32
Growth/differentiation factor 11	GDF11	−1.04	**3.04**	1.68	1.21	1.04
Insulin-like growth factor I	IGF1	**7.10**	**2.70**	**2.35**	1.88	1.14
Insulin-like growth factor II	IGF2	**2.45**	**7.87**	**−3.18**	**−3.43**	−1.21
TGF*β*	TGFB1	1.61	**2.80**	1.71	−1.35	−1.06
TNF*α*	TNFA	1.97	**7.57**	1.67	1.57	**2.10**
